# Crystal structure of bis­(1,3-di­amino­propane-κ^2^ *N*,*N*′)bis­[2-(4-nitro­phen­yl)acetato-κ*O*]zinc(II)

**DOI:** 10.1107/S2056989015022380

**Published:** 2015-12-06

**Authors:** T. J. Roberts, T. F. Mehari, Z. Assefa, T. Hamby, R. E. Sykora

**Affiliations:** a 1601 E Market St., Department of Chemistry, North Carolina, A & T State University, Greensboro, NC 27411, USA; b University of South Alabama, Department of Chemistry, Mobile, AL 36688-0002

**Keywords:** crystal structure, zinc complex, coordination

## Abstract

In the structure of the title compound, [Zn(C_8_H_6_NO_4_)_2_(C_3_H_10_N_2_)_2_], the Zn^II^ atom is located on a center of symmetry with one independent Zn—O distance of 2.199 (2) Å, and two Zn—N distances of 2.157 (2) and 2.144 (2) Å. The overall coordination geometry around the Zn^II^ atom is octa­hedral. Several types of hydrogen-bonding inter­actions are evident. Both intra­molecular [2.959 (3) Å] and inter­molecular [3.118 (3) and 3.124 (3) Å inter­actions occur between the O atoms of the acetate group and the amino N atoms, and weak inter­molecular C—H—O inter­actions involving the nitro groups, leading to an extended chain of the molecules aligned along the *ac* plane.

## Related literature   

For related polymeric Zn^II^ tetra­hedral structural studies, see: Sheng *et al.* (2014 ▸).
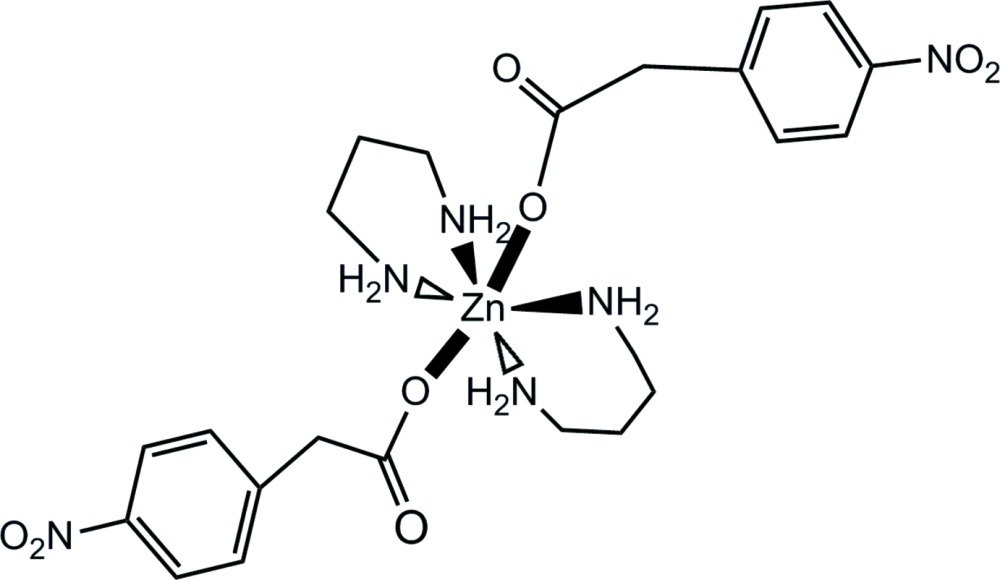


## Experimental   

### Crystal data  

[Zn(C_8_H_6_NO_4_)_2_(C_3_H_10_N_2_)_2_]
*M* *_r_* = 573.91
Monoclinic, 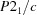

*a* = 14.3933 (18) Å
*b* = 11.0261 (14) Å
*c* = 8.2453 (11) Å
β = 105.119 (13)°
*V* = 1263.3 (3) Å^3^
*Z* = 2
Mo *K*α radiation
μ = 1.03 mm^−1^
*T* = 180 K
0.24 × 0.21 × 0.08 mm

### Data collection  

Agilent Xcalibur, Eos diffractometer
Absorption correction: multi-scan (*CrysAlis PRO*; Agilent, 2013 ▸) *T*_min_ = 0.952, *T*_max_ = 1.000
5782 measured reflections
2243 independent reflections
1858 reflections with *I* > 2σ(*I*)
*R*_int_ = 0.027

### Refinement  

*R*[*F* ^2^ > 2σ(*F* ^2^)] = 0.034
*wR*(*F* ^2^) = 0.075
*S* = 1.06
2243 reflections
169 parameters
H-atom parameters constrained
Δρ_max_ = 0.24 e Å^−3^
Δρ_min_ = −0.34 e Å^−3^

### 

Data collection: *CrysAlis PRO* (Agilent, 2013 ▸); cell refinement: *CrysAlis PRO*; data reduction: *CrysAlis PRO*; program(s) used to solve structure: *Olex2.solve* (Bourhis *et al.*, 2015 ▸); program(s) used to refine structure: *SHELXL97* (Sheldrick, 2008 ▸); molecular graphics: *OLEX2* (Dolomanov *et al.*, 2009 ▸); software used to prepare material for publication: *OLEX2*.

## Supplementary Material

Crystal structure: contains datablock(s) I. DOI: 10.1107/S2056989015022380/hg5460sup1.cif

Supporting information file. DOI: 10.1107/S2056989015022380/hg5460Isup2.cdx

Structure factors: contains datablock(s) I. DOI: 10.1107/S2056989015022380/hg5460Isup3.hkl

**Fig. 1.**
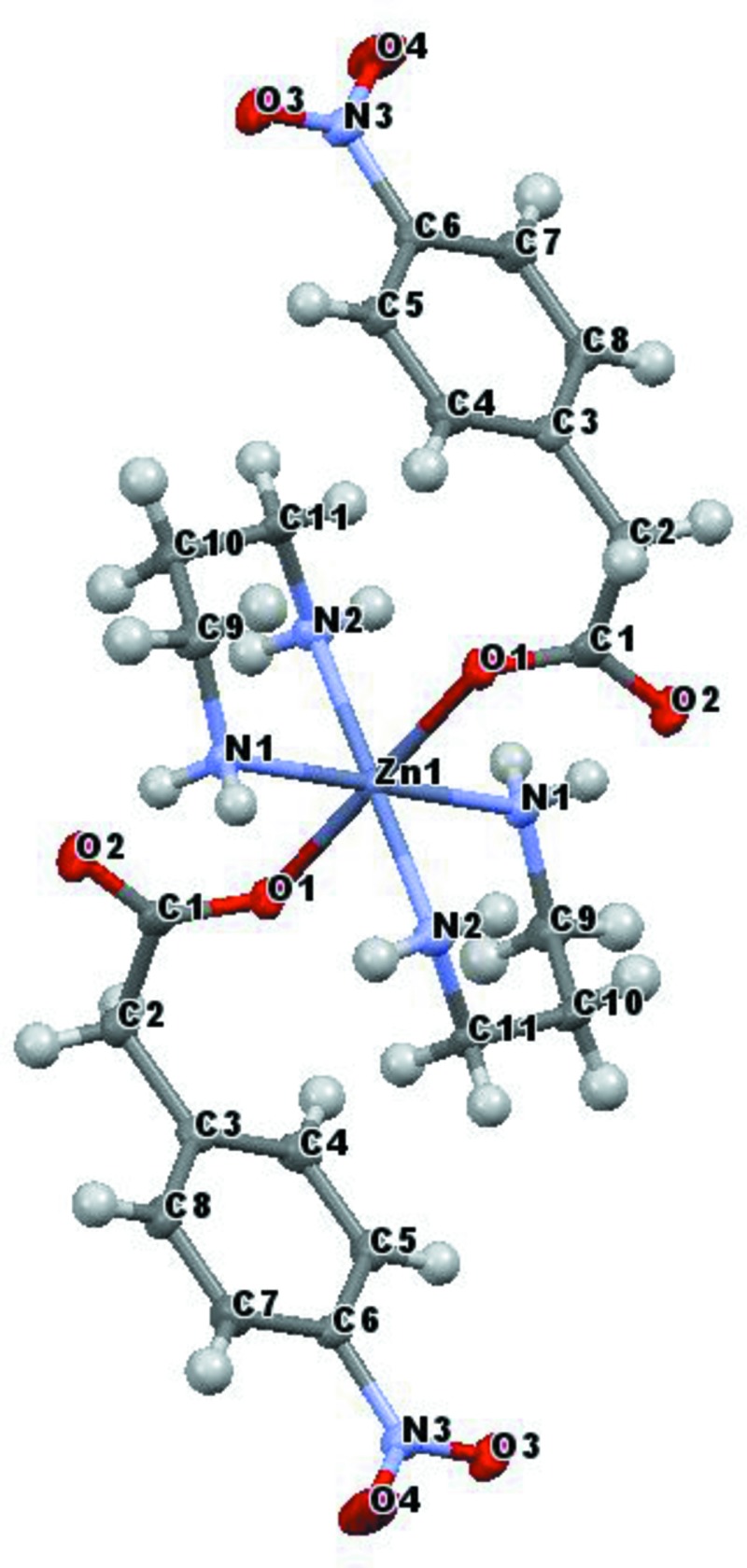
A thermal ellipsoid diagram of the title compound.

. DOI: 10.1107/S2056989015022380/hg5460fig1.tif
A thermal ellipsoid diagram of the title compound.

**Fig. 2.**
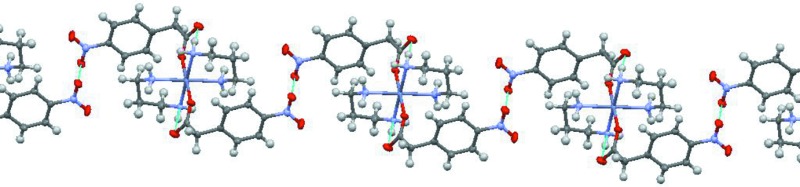
A packing diagram of the title compound.

. DOI: 10.1107/S2056989015022380/hg5460fig2.tif
A packing diagram of the title compound.

CCDC reference: 1438434

Additional supporting information:  crystallographic information; 3D view; checkCIF report

## Figures and Tables

**Table 1:** Hydrogen-bond geometry (Å, °)

*D*—H⋯*A*	*D*—H	H⋯*A*	*D*⋯*A*	*D*—H⋯*A*
N1—H1*B*⋯O2	0.90	2.14	2.959 (3)	150
N1—H1*A*⋯O2^i^	0.90	2.26	3.118 (3)	158
N2—H2*D*⋯O2^ii^	0.90	2.25	3.124 (3)	165
C10—H10*A*⋯O4^iii^	0.97	2.70	3.634 (3)	161
C7—H7⋯O3^iv^	0.93	2.46	3.209 (3)	138

Symmetry codes: (i) 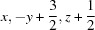; (ii) 
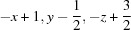
; (iii) 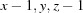; (iv) 
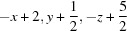
.

## supplementary crystallographic information

### S1. Chemical context

The structure shows a weak N—H···O intra­molecular H-bonding inter­action involving a N atom of the di­amino­propane ligand and an O atom of the nitro­phenyl­acetic acid ligand. Additional inter­molecular N—H···O H-bonding inter­actions involving these same ligands are also present. Several weak inter­molecular H-bonding inter­actions of the C—H—O type also exist involving the nitro groups and neigbouring phenyl protons. The asymmetric unit consist of half of the molecule as the Zn atom is located at center of symmetry.

### S2. Structural commentary

The structure shows a weak H-bond intra­molecular N—H···O inter­action involving the N atoms of the di­amino­propane ligand and the O atoms of nitro­phenyl­acetic acid ligand. Several weak inter­molecular H-bonding inter­actions of the C—H—O type also exist involving the nitro groups and neigbouring C—H protons. The asymmetric unit consist of half of the molecule as the Zn atom is located at center of symmetry.

### S3. Supra­molecular features

H-atoms were placed in calculated positions and allowed to ride during subsequent refinement, with *U*_iso_(H) = 1.2*U*_eq_(C) and C—H distances of 0.93 Å for aromatic hydrogens, *U*_iso_(H) = 1.2*U*_eq_(C) and C—H distances of 0.97 Å for secondary methyl hydrogens, and *U*_iso_(H) = 1.2*U*_eq_(N) and N—H distances of 0.90 Å for amino hydrogens.

### S4. Synthesis and crystallization

Undergraduate student participants enrolled in an open inquiry laboratory (OIL) course conducted the synthesis and charcterization procedures. 0.2 mmol (27.2 mg) of anhydrous ZnCl_2_, 0.4 mmol (29.7 mg) of 1,3-di­amino­propane, and 0.4 mmol (72.5 mg) of 4- nitro­phenyl­acetic acid were weighed and added into a 25-mL round-bottom flask containing 10 mL of 1:1 CH_3_CN/H_2_O mixture. The flask was connected to a condenser and placed on a sand bath and refluxed for 1 hr. The flask was then removed from the sandbath and cooled to room temperature. After filtering the solution, the clear supernatant was collected, and covered with a parafilm for slow evaporation. Single crystals suitable for X-ray measurement were found after several days.

### S5. Refinement

Crystal data, data collection and structure refinement details are summarized in Table 1.

### Crystal data

**Table:**

[Zn(C_8_H_6_NO_4_)_2_(C_3_H_10_N_2_)_2_]	*F*(000) = 600
*M**_r_* = 573.91	*D*_x_ = 1.509 Mg m^−^^3^
Monoclinic, *P*2_1_/*c*	Mo *K*α radiation, λ = 0.71073 Å
*a* = 14.3933 (18) Å	Cell parameters from 2201 reflections
*b* = 11.0261 (14) Å	θ = 3.2–27.2°
*c* = 8.2453 (11) Å	µ = 1.03 mm^−^^1^
β = 105.119 (13)°	*T* = 180 K
*V* = 1263.3 (3) Å^3^	, colourless
*Z* = 2	0.24 × 0.21 × 0.08 mm

### Data collection

**Table:**

Agilent Xcalibur, Eos diffractometer	2243 independent reflections
Radiation source: Enhance (Mo) X-ray Source	1858 reflections with *I* > 2σ(*I*)
Graphite monochromator	*R*_int_ = 0.027
Detector resolution: 16.0514 pixels mm^-1^	θ_max_ = 25.1°, θ_min_ = 2.9°
ω scans	*h* = −16→17
Absorption correction: multi-scan (*CrysAlis PRO*; Agilent, 2013)	*k* = −13→12
*T*_min_ = 0.952, *T*_max_ = 1.000	*l* = −9→9
5782 measured reflections	

### Refinement

**Table:**

Refinement on *F*^2^	Primary atom site location: iterative
Least-squares matrix: full	Secondary atom site location: difference Fourier map
*R*[*F*^2^ > 2σ(*F*^2^)] = 0.034	Hydrogen site location: inferred from neighbouring sites
*wR*(*F*^2^) = 0.075	H-atom parameters constrained
*S* = 1.06	*w* = 1/[σ^2^(*F*_o_^2^) + (0.0245*P*)^2^ + 0.7294*P*] where *P* = (*F*_o_^2^ + 2*F*_c_^2^)/3
2243 reflections	(Δ/σ)_max_ < 0.001
169 parameters	Δρ_max_ = 0.24 e Å^−^^3^
0 restraints	Δρ_min_ = −0.34 e Å^−^^3^

### Special details

**Table:**

Geometry. All e.s.d.'s (except the e.s.d. in the dihedral angle between two l.s. planes) are estimated using the full covariance matrix. The cell e.s.d.'s are taken into account individually in the estimation of e.s.d.'s in distances, angles and torsion angles; correlations between e.s.d.'s in cell parameters are only used when they are defined by crystal symmetry. An approximate (isotropic) treatment of cell e.s.d.'s is used for estimating e.s.d.'s involving l.s. planes.
Refinement. Refinement of *F*^2^ against ALL reflections. The weighted *R*-factor *wR* and goodness of fit *S* are based on *F*^2^, conventional *R*-factors *R* are based on *F*, with *F* set to zero for negative *F*^2^. The threshold expression of *F*^2^ > σ(*F*^2^) is used only for calculating *R*-factors(gt) *etc*. and is not relevant to the choice of reflections for refinement. *R*-factors based on *F*^2^ are statistically about twice as large as those based on *F*, and *R*- factors based on ALL data will be even larger.

### Fractional atomic coordinates and isotropic or equivalent isotropic displacement parameters (Å^2^)

**Table:**

	*x*	*y*	*z*	*U*_iso_*/*U*_eq_	
Zn1	0.5000	0.5000	1.0000	0.01900 (13)	
N3	1.03741 (14)	0.5615 (2)	1.3353 (2)	0.0256 (5)	
O3	1.06699 (12)	0.46346 (16)	1.3979 (2)	0.0292 (4)	
O2	0.57083 (13)	0.64366 (17)	0.6651 (2)	0.0351 (5)	
C1	0.63039 (17)	0.5747 (2)	0.7576 (3)	0.0205 (6)	
C4	0.84282 (17)	0.4574 (2)	0.9699 (3)	0.0240 (6)	
H4	0.8148	0.3851	0.9235	0.029*	
C3	0.81098 (16)	0.5666 (2)	0.8894 (3)	0.0197 (5)	
O1	0.61567 (11)	0.50684 (15)	0.8709 (2)	0.0243 (4)	
O4	1.07279 (15)	0.65867 (17)	1.3915 (2)	0.0482 (6)	
C6	0.95606 (16)	0.5630 (2)	1.1840 (3)	0.0196 (5)	
C8	0.85334 (17)	0.6733 (2)	0.9612 (3)	0.0244 (6)	
H8	0.8323	0.7468	0.9094	0.029*	
C2	0.73151 (16)	0.5702 (2)	0.7286 (3)	0.0240 (6)	
H2A	0.7364	0.4989	0.6624	0.029*	
H2B	0.7404	0.6409	0.6642	0.029*	
C5	0.91535 (17)	0.4548 (2)	1.1176 (3)	0.0220 (6)	
H5	0.9362	0.3817	1.1710	0.026*	
C7	0.92621 (18)	0.6726 (2)	1.1082 (3)	0.0258 (6)	
H7	0.9545	0.7447	1.1551	0.031*	
N2	0.40460 (13)	0.42319 (18)	0.7777 (2)	0.0230 (5)	
H2C	0.4313	0.4334	0.6913	0.028*	
H2D	0.4002	0.3429	0.7937	0.028*	
N1	0.45008 (13)	0.67537 (17)	0.9027 (2)	0.0210 (5)	
H1A	0.4698	0.7295	0.9863	0.025*	
H1B	0.4800	0.6941	0.8227	0.025*	
C11	0.30623 (16)	0.4742 (2)	0.7291 (3)	0.0222 (6)	
H11A	0.2767	0.4641	0.8214	0.027*	
H11B	0.2679	0.4296	0.6334	0.027*	
C9	0.34572 (17)	0.6935 (2)	0.8304 (3)	0.0273 (6)	
H9A	0.3340	0.7765	0.7918	0.033*	
H9B	0.3122	0.6800	0.9168	0.033*	
C10	0.30620 (18)	0.6076 (2)	0.6844 (3)	0.0277 (6)	
H10A	0.2406	0.6316	0.6301	0.033*	
H10B	0.3437	0.6178	0.6032	0.033*	

### Atomic displacement parameters (Å^2^)

**Table:**

	*U*^11^	*U*^22^	*U*^33^	*U*^12^	*U*^13^	*U*^23^
Zn1	0.0173 (2)	0.0156 (2)	0.0208 (2)	−0.00001 (18)	−0.00090 (15)	0.00156 (17)
N3	0.0224 (11)	0.0271 (13)	0.0249 (12)	0.0014 (11)	0.0017 (9)	0.0007 (10)
O3	0.0300 (10)	0.0269 (10)	0.0277 (10)	0.0089 (9)	0.0020 (8)	0.0051 (8)
O2	0.0287 (10)	0.0400 (12)	0.0358 (11)	0.0111 (10)	0.0069 (8)	0.0188 (9)
C1	0.0219 (12)	0.0209 (14)	0.0165 (12)	−0.0026 (12)	0.0010 (10)	−0.0045 (11)
C4	0.0234 (13)	0.0198 (14)	0.0287 (14)	−0.0019 (12)	0.0068 (11)	−0.0044 (11)
C3	0.0165 (12)	0.0248 (14)	0.0204 (13)	0.0009 (11)	0.0094 (10)	0.0001 (11)
O1	0.0208 (9)	0.0283 (10)	0.0247 (9)	0.0035 (8)	0.0074 (7)	0.0097 (8)
O4	0.0500 (13)	0.0269 (11)	0.0486 (12)	−0.0097 (11)	−0.0210 (10)	−0.0022 (9)
C6	0.0162 (12)	0.0217 (14)	0.0204 (13)	0.0009 (11)	0.0038 (10)	0.0010 (11)
C8	0.0264 (13)	0.0195 (14)	0.0259 (14)	0.0061 (12)	0.0043 (11)	0.0046 (11)
C2	0.0225 (13)	0.0310 (15)	0.0190 (13)	−0.0002 (12)	0.0061 (10)	0.0012 (11)
C5	0.0223 (13)	0.0174 (13)	0.0263 (14)	0.0013 (11)	0.0064 (11)	0.0027 (11)
C7	0.0283 (14)	0.0186 (13)	0.0285 (14)	−0.0025 (12)	0.0039 (11)	−0.0022 (11)
N2	0.0236 (11)	0.0223 (12)	0.0224 (11)	−0.0005 (10)	0.0047 (9)	−0.0013 (9)
N1	0.0235 (11)	0.0194 (11)	0.0182 (10)	−0.0007 (10)	0.0020 (8)	0.0002 (9)
C11	0.0203 (12)	0.0263 (15)	0.0167 (12)	−0.0049 (11)	−0.0012 (10)	−0.0007 (10)
C9	0.0245 (14)	0.0204 (14)	0.0335 (15)	0.0062 (12)	0.0011 (11)	0.0008 (11)
C10	0.0217 (13)	0.0290 (15)	0.0262 (14)	0.0012 (12)	−0.0047 (11)	0.0050 (11)

### Geometric parameters (Å, º)

**Table:**

Zn1—O1	2.1989 (16)	C8—C7	1.381 (3)
Zn1—O1^i^	2.1989 (16)	C2—H2A	0.9700
Zn1—N2	2.1565 (18)	C2—H2B	0.9700
Zn1—N2^i^	2.1565 (18)	C5—H5	0.9300
Zn1—N1^i^	2.1444 (19)	C7—H7	0.9300
Zn1—N1	2.1444 (19)	N2—H2C	0.9000
N3—O3	1.226 (3)	N2—H2D	0.9000
N3—O4	1.224 (3)	N2—C11	1.478 (3)
N3—C6	1.472 (3)	N1—H1A	0.9000
O2—C1	1.246 (3)	N1—H1B	0.9000
C1—O1	1.257 (3)	N1—C9	1.478 (3)
C1—C2	1.536 (3)	C11—H11A	0.9700
C4—H4	0.9300	C11—H11B	0.9700
C4—C3	1.394 (3)	C11—C10	1.517 (3)
C4—C5	1.382 (3)	C9—H9A	0.9700
C3—C8	1.384 (3)	C9—H9B	0.9700
C3—C2	1.509 (3)	C9—C10	1.520 (3)
C6—C5	1.378 (3)	C10—H10A	0.9700
C6—C7	1.377 (3)	C10—H10B	0.9700
C8—H8	0.9300		
			
O1^i^—Zn1—O1	180.00 (9)	C3—C2—H2B	108.9
N2—Zn1—O1^i^	90.20 (7)	H2A—C2—H2B	107.7
N2^i^—Zn1—O1	90.20 (7)	C4—C5—H5	120.8
N2—Zn1—O1	89.80 (7)	C6—C5—C4	118.5 (2)
N2^i^—Zn1—O1^i^	89.80 (7)	C6—C5—H5	120.8
N2^i^—Zn1—N2	180.0	C6—C7—C8	118.6 (2)
N1^i^—Zn1—O1	89.43 (7)	C6—C7—H7	120.7
N1—Zn1—O1	90.57 (7)	C8—C7—H7	120.7
N1—Zn1—O1^i^	89.43 (7)	Zn1—N2—H2C	108.4
N1^i^—Zn1—O1^i^	90.57 (7)	Zn1—N2—H2D	108.4
N1—Zn1—N2	87.65 (7)	H2C—N2—H2D	107.4
N1^i^—Zn1—N2^i^	87.65 (7)	C11—N2—Zn1	115.69 (14)
N1^i^—Zn1—N2	92.35 (7)	C11—N2—H2C	108.4
N1—Zn1—N2^i^	92.35 (7)	C11—N2—H2D	108.4
N1^i^—Zn1—N1	180.0	Zn1—N1—H1A	107.7
O3—N3—C6	118.6 (2)	Zn1—N1—H1B	107.7
O4—N3—O3	123.2 (2)	H1A—N1—H1B	107.1
O4—N3—C6	118.2 (2)	C9—N1—Zn1	118.60 (15)
O2—C1—O1	126.5 (2)	C9—N1—H1A	107.7
O2—C1—C2	116.9 (2)	C9—N1—H1B	107.7
O1—C1—C2	116.5 (2)	N2—C11—H11A	109.2
C3—C4—H4	119.4	N2—C11—H11B	109.2
C5—C4—H4	119.4	N2—C11—C10	112.0 (2)
C5—C4—C3	121.1 (2)	H11A—C11—H11B	107.9
C4—C3—C2	121.4 (2)	C10—C11—H11A	109.2
C8—C3—C4	118.5 (2)	C10—C11—H11B	109.2
C8—C3—C2	120.1 (2)	N1—C9—H9A	109.3
C1—O1—Zn1	132.51 (15)	N1—C9—H9B	109.3
C5—C6—N3	119.3 (2)	N1—C9—C10	111.5 (2)
C7—C6—N3	118.6 (2)	H9A—C9—H9B	108.0
C7—C6—C5	122.0 (2)	C10—C9—H9A	109.3
C3—C8—H8	119.3	C10—C9—H9B	109.3
C7—C8—C3	121.3 (2)	C11—C10—C9	115.8 (2)
C7—C8—H8	119.3	C11—C10—H10A	108.3
C1—C2—H2A	108.9	C11—C10—H10B	108.3
C1—C2—H2B	108.9	C9—C10—H10A	108.3
C3—C2—C1	113.32 (19)	C9—C10—H10B	108.3
C3—C2—H2A	108.9	H10A—C10—H10B	107.4
			
Zn1—N2—C11—C10	63.2 (2)	O4—N3—C6—C7	2.9 (3)
Zn1—N1—C9—C10	−59.0 (2)	C8—C3—C2—C1	−92.6 (3)
N3—C6—C5—C4	−176.2 (2)	C2—C1—O1—Zn1	166.11 (15)
N3—C6—C7—C8	176.5 (2)	C2—C3—C8—C7	−180.0 (2)
O3—N3—C6—C5	0.2 (3)	C5—C4—C3—C8	−0.3 (4)
O3—N3—C6—C7	−176.5 (2)	C5—C4—C3—C2	−179.7 (2)
O2—C1—O1—Zn1	−15.1 (4)	C5—C6—C7—C8	−0.1 (4)
O2—C1—C2—C3	138.9 (2)	C7—C6—C5—C4	0.4 (4)
C4—C3—C8—C7	0.7 (4)	N2—Zn1—O1—C1	74.1 (2)
C4—C3—C2—C1	86.7 (3)	N2^i^—Zn1—O1—C1	−105.9 (2)
C3—C4—C5—C6	−0.2 (4)	N2^i^—Zn1—N1—C9	−135.43 (17)
C3—C8—C7—C6	−0.4 (4)	N2—Zn1—N1—C9	44.58 (17)
O1^i^—Zn1—N2—C11	43.83 (16)	N2—C11—C10—C9	−70.8 (3)
O1—Zn1—N2—C11	−136.17 (16)	N1^i^—Zn1—O1—C1	166.4 (2)
O1^i^—Zn1—N1—C9	−45.65 (17)	N1—Zn1—O1—C1	−13.6 (2)
O1—Zn1—N1—C9	134.35 (17)	N1^i^—Zn1—N2—C11	134.41 (16)
O1—C1—C2—C3	−42.2 (3)	N1—Zn1—N2—C11	−45.59 (16)
O4—N3—C6—C5	179.6 (2)	N1—C9—C10—C11	67.2 (3)

Symmetry code: (i) −*x*+1, −*y*+1, −*z*+2.

### Hydrogen-bond geometry (Å, º)

**Table:**

*D*—H···*A*	*D*—H	H···*A*	*D*···*A*	*D*—H···*A*
N1—H1*B*···O2	0.90	2.14	2.959 (3)	150
N1—H1*A*···O2^ii^	0.90	2.26	3.118 (3)	158
N2—H2*D*···O2^iii^	0.90	2.25	3.124 (3)	165
C10—H10*A*···O4^iv^	0.97	2.70	3.634 (3)	161
C7—H7···O3^v^	0.93	2.46	3.209 (3)	138

Symmetry codes: (ii) *x*, −*y*+3/2, *z*+1/2; (iii) −*x*+1, *y*−1/2, −*z*+3/2; (iv) *x*−1, *y*, *z*−1; (v) −*x*+2, *y*+1/2, −*z*+5/2.
