# Supplementary figures and images for: Crystal structure of bis­(1,3-di­amino­propane-κ2 N,N′)bis­[2-(4-nitro­phen­yl)acetato-κO]zinc(II)
Source: Acta Crystallogr E Crystallogr Commun. 2015 Dec 6;71(Pt 12):m240–1. doi: 10.1107/S2056989015022380 (PMC4719853; doi:10.1107/S2056989015022380)

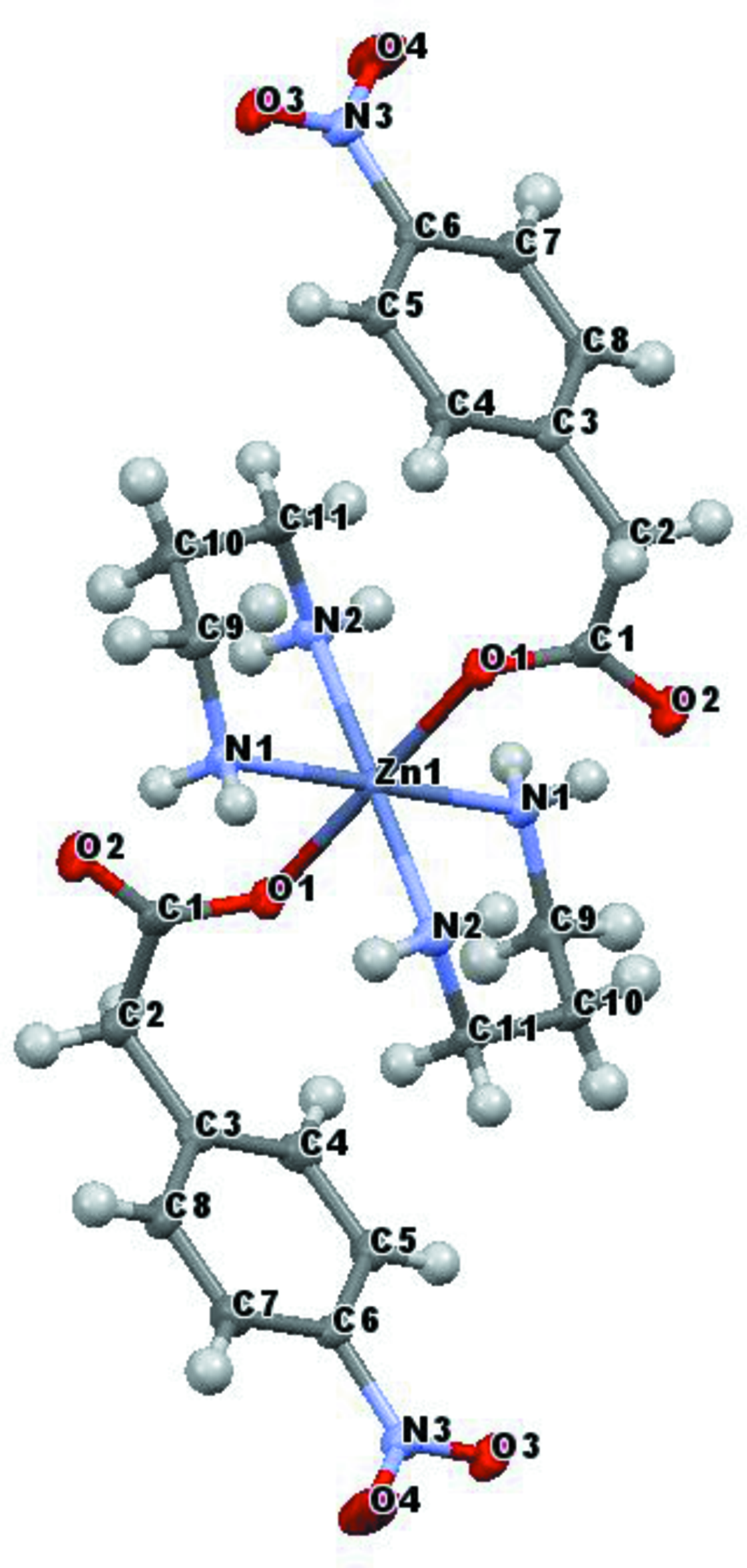

Supplement: Supplementary file 4 [file e-71-0m240-fig1.tif]

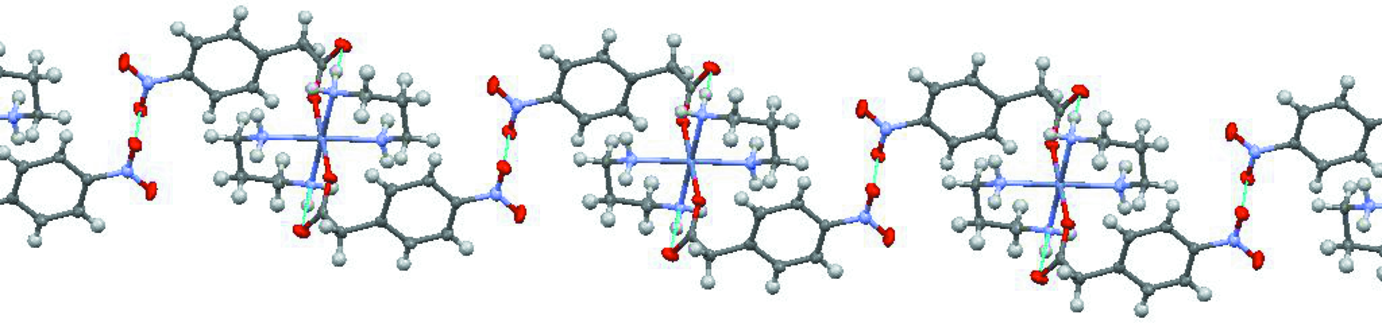

Supplement: Supplementary file 5 [file e-71-0m240-fig2.tif]
